# Uncovering the function of insulin receptor substrate in termites’ immunity through active immunization

**DOI:** 10.1093/jisesa/ieae061

**Published:** 2024-07-03

**Authors:** Wei Zhou, Xingying Zhao, Ali Hassan, Bao Jia, Long Liu, Qiuying Huang

**Affiliations:** Hubei Insect Resources Utilization and Sustainable Pest Management Key Laboratory, Huazhong Agricultural University, Wuhan 430070, China; Hubei Insect Resources Utilization and Sustainable Pest Management Key Laboratory, Huazhong Agricultural University, Wuhan 430070, China; Hubei Insect Resources Utilization and Sustainable Pest Management Key Laboratory, Huazhong Agricultural University, Wuhan 430070, China; Hubei Insect Resources Utilization and Sustainable Pest Management Key Laboratory, Huazhong Agricultural University, Wuhan 430070, China; Nanning Institute of Termite Control, Nanning 530023, China; Henan International Laboratory for Green Pest Control, Henan Engineering Laboratory of Pest Biological Control, College of Plant Protection, Henan Agricultural University, Zhengzhou 450002, China; Hubei Insect Resources Utilization and Sustainable Pest Management Key Laboratory, Huazhong Agricultural University, Wuhan 430070, China

**Keywords:** insulin signaling, *Reticulitermes chinensis*, *Metarhizium anisopliae*, social immunity, survival

## Abstract

Insulin receptor substrate (IRS) proteins are key mediators in insulin signaling pathway. In social insect lives, IRS proteins played important roles in caste differentiation and foraging, but there function in disease defenses such as active immunization has not been reported yet. To investigate the issue, we successfully suppressed the *IRS* gene 3 days after dsRNA injection. Suppressing *IRS* gene increased the contents of glucose, trehalose, glycogen, and triglyceride and decreased the content of pyruvate in termites, and led to the metabolic disorder of glucose and lipids. *IRS* suppressing significantly enhanced grooming behaviors of nestmates of fungus-contaminated termites and hence increased the conidial load in the guts of the nestmates. Additionally, *IRS* suppressing led to significant downregulation of the immune genes *Gram-negative bacteria-binding protein2* (GNBP2) and *termicin* and upregulation of the apoptotic gene *caspase8*, and hence diminished antifungal activity of nestmates of fungus-contaminated termites. The above abnormal behavioral and physiological responses significantly decreased the survival rate of ds*IRS*-injected nestmates of the fungus-contaminated termites. These findings suggest that IRS is involved in regulation of active immunization in termites, providing a better understanding of the link between insulin signaling and the social immunity of termites.

## Introduction

In social insect colonies, pathogenic infections have the potential to spread rapidly and cause disease outbreaks (Pull et al. 2018). However, social insects have the ability to protect themselves by utilizing individual defenses and colony-wide systematic responses against pathogens, providing the colony with protection known as “social immunity” (Cremer 2019, Liu et al. 2019). Social immunity includes a series of immune strategies such as chemical communication, hygienic behavior, and physiological immune response to resist infections by pathogens at individual and colony levels (Liu et al. 2015, Xiong et al. 2019, Hassan et al. 2021). Grooming behavior is one of the most important behavioral immunities, by which nestmates can remove infectious particles from the body surfaces of exposed individuals (Tragust et al. 2013, Theis et al. 2015, Zhou et al. 2021). Pathogens are transferred from pathogen-exposed insects to the nestmates and cause low-level infections during grooming (Shimizu and Yamaji 2003, Yanagawa and Shimizu 2007). Low-level infections rarely result in death but instead upregulate immune genes and hence promote an enhanced ability to inhibit growth of pathogens (Konrad et al. 2012). Individuals in the colony get immunization against a specific pathogen during infection process, which helps to fight against the same pathogens in the future, this process is termed as active immunization (Masri and Cremer 2014, Liu et al. 2015). Distinguished from passive immunity, which relies on acquiring immune substances to enhance disease resistance, active immunity activates the host’s own immune system (Konrad et al. 2012, Masri and Cremer 2014).

As a key regulator of glucose homeostasis, insulin plays important anabolic functions throughout the body and is involved in regulating the growth, reproduction, and caste differentiation of social insects (Corona et al. 2016). The insulin signaling pathway also regulates the aging of termite *Reticulitermes chinensis* (Snyder) (Haroon et al. 2021). Insulin signaling can affect the metabolism of the organism and promote the storage of carbohydrates and lipids in *Drosophila*, which is reflected in its ability to control the adipocyte cell number and triglyceride storage (DiAngelo et al. 2009). Defects in the insulin/insulin-like growth factor signaling pathway manifest as a collection of metabolic conditions in *Drosophila* (Das and Dobens 2015). Modulation of insulin receptor substrate (IRS) was sufficient to alter the developmental fate of a queen-destined larva into a worker phenotype in honeybees *Apis mellifera* (Mutti et al. 2011, Wolschin et al. 2011). A study of the ant *Diacamma* sp. found that the asymmetry in reproductive potential between ants was correlated with insulin receptor expression in the ovaries (Okada et al. 2010). The interplay between vitellogenin, juvenile hormone, and insulin signaling regulates queen lifespan of honeybees *A. mellifera* without sacrificing fecundity (Corona et al., 2007). Expression of peripheral *IRS* can also affect food choice in foraging behavior of the honeybee *A. mellifera* (Wang et al. 2010). Insulin signaling is involved in the regulation of worker division of labor in honeybee colonies and soldier-specific morphological changes in termite *Hodotermopsis sjostedti* (Hattori et al. 2013). The insulin receptor holds potential as an important target for the development of new drugs and pesticides, given its physiological effects and molecular mechanisms (Zhang et al. 2022). These studies show the close relationship between insulin signaling and social insect lives. However, as social immunity is also an important aspect of social insect lives, whether insulin signaling participates in such colony-level disease defenses is still unclear.

The subterranean termite *R. chinensis* Snyder is an important pest of forest trees and urban buildings in China, including Beijing, Tianjin, Shanxi, and the Yangtze River drainage basin (Huang et al. 2013, Gao et al. 2018). The entomopathogenic fungus (EPF) *Metarhizium anisopliae* (Metchnikoff) Sorokin is an ideal experimental fungus for studying insect social immunity and is being developed in biological pest control (Masri and Cremer 2014, Liu et al. 2019). Here, we used *R. chinensis* and *M. anisopliae* as the tested materials to determine the effect of insulin signaling on active immunization in termites. To address it, we suppressed the *IRS* gene and then detected the changes of metabolites (glucose, trehalose, glycogen, pyruvate, and triglyceride) in termites, and tested the behavioral immunity (grooming behavior and conidial load) and physiological immunity (antifungal activity and immune-related gene expressions) in nestmates of the fungus-contaminated termites. We further tested the survival rate of ds*IRS*-injected nestmates of fungus-contaminated termites so as to verify the effect of *IRS*-mediated disruption of active immunization on the resistance of termite groups to fungal infections. This study will offer a new avenue to link insulin signaling pathway to active immunization in termites, and will help to build a theoretical basis for deeply revealing the social immunity of termites and other social insects.

## Materials and Methods

### Experimental Termites

Worker termites were collected from the 9 *R. chinensis* colonies in Shizi Hill, Wuhan City, Hubei Province, China. The termites from each colony were reared in a plastic container (25 cm × 15 cm × 10 cm) under laboratory conditions of 25 ± 1 °C, 80 ± 5% RH, and 24 h of darkness. We used pine as the food source for termites. Healthy adult workers were chosen for the subsequent experiments.

### Fungal Pathogens

The *R. chinensis* workers were contaminated with the EPF *M. anisopliae* (IBCCM321.93). *M. anisopliae* was cultivated on potato dextrose agar (PDA) (Rishui, China), and plates were placed upside down in an artificial climate box that maintained a constant temperature of 25 °C for approximately 10 days. The spores of *M. anisopliae* were collected with 0.1% Tween 80 and stored in a refrigerator at 4 °C. The spore germination rate used in all the experiments was greater than 90% (Liu et al. 2019). In the fungal treatment group of subsequent experiments, we followed the previously published protocol (Cremer 2019) by pipetting 0.35 μl of the spore suspension (10^8^ conidia/ml) onto the tergites of the termite abdomen. The termites were then placed into a 4 °C refrigerator for 30 min to reduce movement, allowing the conidia to attach to their body surface (Traniello et al. 2002). In the control group, 0.35 μl of sterile 0.1% Tween 80 solution was placed on the tergites of the termite abdomen and placed in 4 °C refrigerator for 30 min.

### Cloning and Phylogenetic Analysis of the *IRS* Gene

Total RNA was extracted from 3 *R. chinensis* workers using TRIzol (Takara, Japan). The quality and concentration of the extracted RNA were measured with a Nanodrop 2000 spectrophotometer (Thermo Fisher Scientific, USA). We synthesized cDNA from 500 ng of total RNA by using the PrimeScript RT Reagent Kit with gDNA Eraser (Perfect Real Time) (Takara, Japan) according to the manufacturer’s instructions. The primers for gene amplification (Supplementary Table S1) were designed based on the transcriptome database of *R. chinensis* (unpublished). The polymerase chain reaction (PCR) products were purified by using the AxyPrep DNA Gel Extraction Kit (Axygen Scientific, USA), cloned into the pMD18-T (Takara, Japan), transformed by using Trans1-T1 Phage Resistant Chemically Competent Cells (Transgen Biotech, China), and sequenced by Tsingke Biological Technology (China). The results were analyzed by NCBI BLAST (National Center for Biotechnology Information) (http://www.ncbi.nlm.nih.gov/). Primers based on the sequencing results were designed to verify the size of the target gene (Supplementary Table S1). The protein alignment and phylogenetic analysis were performed using MEGA 7 software. The multiple sequence alignment was conducted with the ClustalW algorithm, and the phylogenetic tree was constructed using the Neighbor-Joining method. The reliability of the tree structure was assessed using a bootstrap procedure based on 1,000 replicates.

### *IRS* Gene Expression in Active Immunization

One fungus-contaminated termite and 5 naïve nestmates were placed together in a petri dish (*D* = 3.5 cm) with moist filter paper at the bottom. The head of the fungus-contaminated termite was marked with a black marker. After 1 day, 3 nestmates of the fungus-contaminated termites were randomly selected from each petri dish and placed in a 1.5-ml centrifuge tube to extract the total RNA. The nestmates of the Tween 80-treated termites were used as the control samples. There were 9 replications from 3 termite colonies, each colony with 3 replications.

Approximately 500 ng of RNA was converted to cDNA. RT-qPCR primers were designed using NCBI (https://www.ncbi.nlm.nih.gov/tools/primer-blast) (Supplementary Table S1) and synthesized by Tsingke Biological Technology (China). RT-qPCR was performed in a Bio-Rad CFX Connect Real-Time PCR System (Bio-Rad, USA). The PCR conditions consisted of 3 min at 95 °C followed by 40 cycles of 10 s at 95 °C and 30 s at 58 °C. The reaction mixtures were prepared according to the manufacturer’s protocol (Yeasen, China). The relative expression level of the IRS gene was normalized to the expression level of Heat Shock Protein 70 (*HSP70*) and *β-actin*, which were used as reference genes, with the 2^−ΔΔCT^ method (Livak&Schmittgen 2001).

### RNAi Efficiency after Injecting ds*IRS*

To verify the function of *IRS* gene in active immunization of termites, we synthesized ds*IRS* and estimated the RNAi efficiency of ds*IRS*. Primers (Supplementary Table S1) containing the T7 RNA polymerase promoter were used to amplify ds*GFP* and ds*IRS* by PCR. The dsRNA was synthesized and purified according to the manufacturer’s instructions (Thermo Fisher Scientific, USA). Purity of the extracted dsRNA was checked through Nanodrop 2000 spectrophotometer (Thermo Fisher Scientific, USA).

We injected 100 nl (2 μg) dsRNA between the second and third thoracic segment of the termites with a microinjector (PV820, WPI Inc, Germany) (Zhou et al. 2006). After injection, the termites were placed in a plastic petri dish (*D* = 9 cm) with wet filter paper at the bottom and reared for 3 days. The efficiency of RNAi was detected by RT-qPCR. The termites injected with double-stranded *IRS* were called as the ds*IRS*-treated group, and the termites injected with double-stranded *green fluorescent protein* (*GFP*) were called as the ds*GFP*-treated group (the control). There were 9 replications from 3 termite colonies, each colony with 3 replications.

### Determination of Metabolic Substances

The content of all metabolites was determined by using available assay kits (Nanjing Jiancheng Bioengineering Institute, China) based on the manufacturer’s protocols of these kits. We determined the levels of glucose, trehalose, glycogen, pyruvate, and triglyceride in termites injected with ds*IRS* and ds*GFP* using the GPO-PAP method at respective wavelengths of 505, 620, 620, 505, and 510 nm. There were 9 replications from 3 termite colonies, each colony with 3 replications.

### Behavioral Immunophenotypes

We investigated the grooming behavior changes of ds*IRS*-injected nestmates toward infected individuals. Three days after dsRNA (ds*IRS* or ds*GFP*) injection, 5 dsRNA-injected nestmates were placed in a petri dish (*D* = 3.5 cm) with 1 fungus-contaminated termite. The head of the fungus-contaminated termite was marked with a black marker. To examine whether suppressing *IRS* affected the grooming behavior of the termites, a camera (acA1920-40gc, Basler) was used to continuously record the grooming behavior of termites for 40 min. EthoVision 14.0 tracking software (Noldus Information Technology, Netherlands) was used to analyze the frequency and cumulative time of grooming behavior during which the nestmates groomed the infected individuals. There were 9 replications from 3 termite colonies, each colony with 3 replications.

After 40 min of grooming behavior, the termites injected with ds*IRS* or ds*GFP* were dissected, and their guts were obtained and placed in 50 μl of 0.1% Tween 80, respectively. The guts were disrupted by a glass grinding rod, transferred to PDA selection medium (containing 100 μg/ml streptomycin, 100 μg/ml chloramphenicol, and 100 μg/ml kanamycin), and then placed in 25 ± 1 °C incubator for 1 wk so as to count the number of colony-forming units (CFUs) of *M. anisopliae* in the guts. There were 5 replications from 2 termite colonies.

### Physiological Immunophenotypes

Socially transferred conidia may activate the immune response of the hosts to enhance their antifungal activity, so we analyzed the alterations of the nestmates’ antifungal activity after injection of ds*IRS*. Two days after dsRNA (ds*IRS* or ds*GFP*) injection, 5 dsRNA-injected nestmates were reared in a petri dish (*D* = 3.5 cm) with 1 fungus-contaminated termite for 1 day. The 5 dsRNA-injected nestmates of the fungus-contaminated termites were ground into powder, dissolved in 0.9% NaCl, and subsequently centrifuged to obtain the supernatant. We used 96-well microplates to measure the antifungal activity. We added 50 μl of Sabouraud Dextrose Broth (SDB) and 4 μl of 0.9% NaCl for the standards and 50 μl of SDB, 2 μl of 0.9% NaCl, and 2 μl of *M. anisopliae* spore suspension for the growth control. We added 50 μl of SDB, 2 μl of sample supernatant, and 2 μl of *M. anisopliae* spore suspension for the samples. The microplates were incubated in a constant temperature oscillator for 24 h (25 °C, 200 rpm). The absorbance of each well was measured with a microplate reader (SPAPK, TECAN, Switzerland) at a wavelength of 600 nm every hour to calculate the fungal growth inhibition rate in order to compare the changes in the antifungal activity. Fungal growth inhibition rate = (OD_growth control_ − OD_sample_)/(OD_growth control_ − OD_standard_) (Zhao et al. 2020). There were 9 replications from 3 termite colonies, each colony with 3 replications.

Socially transferred conidia may also improve immune gene expression in hosts, so we examined how the expression of the immune gene *Gram-negative bacteria-binding protrin2* (*GNBP2*), *termicin*, and apoptosis gene *caspase8*, changed in the nestmates after injection of ds*IRS*. Two days after ds*IRS* or ds*GFP* injection, 5 dsRNA-injected nestmates were reared in a petri dish (*D* = 3.5 cm) with 1 fugus-contaminated termite for 1 day. RT-qPCR primers were designed using NCBI (https://www.ncbi.nlm.nih.gov/tools/primer-blast) (Supplementary Table S1). We used RT-qPCR and the 2^−ΔΔCT^ method (Livak and Schmittgen 2001) to determine the change in the expression of the genes *GNBP2*, *termicin*, and *caspase8*. There were 9 replications from 3 termite colonies, each colony with 3 replications.

### Survival

To explore whether suppressing *IRS* can improve the lethal effect of *M. anisopliae* on termites, we observed the changes in the survival of the nestmate termites reared with 1 fungus-contaminated termite after injection of ds*IRS.* Two days after ds*IRS* or ds*GFP* injection, 5 dsRNA-injected nestmates were reared in a petri dish (*D* = 3.5 cm) with 1 fugus-contaminated termite for 20 days. The death of the nestmate termites in the petri dish was recorded every day, and dead termites were removed in a timely manner. The control groups included ds*IRS-* or ds*GFP-*injected termites reared with 1 Tween 80-treated termite. There were 6 replications from 3 termite colonies, each replication had 5 workers used to count the survival rate, and a total of 120 termites were used in this experiment.

### Statistical Analysis

First, we used the Shapiro–Wilk test to detect whether the data conformed to a normal distribution. Data that did not conform to a normal distribution were analyzed for the significance of differences using the Wilcoxon test. The data that obeyed the normal distribution were analyzed for significant differences using independent *t*-tests. Cox proportional regression was used to analyze the effect of termite colonies and experimental treatments on the survival of the termites, and then we used Kaplan–Meier methods to analyze the significant differences in the survival of the termites between the different experimental treatments.

## Results

### Cloning, Expression, and RNAi Efficiency of *IRS* in *R. chinensis*

**Fig. 1.**
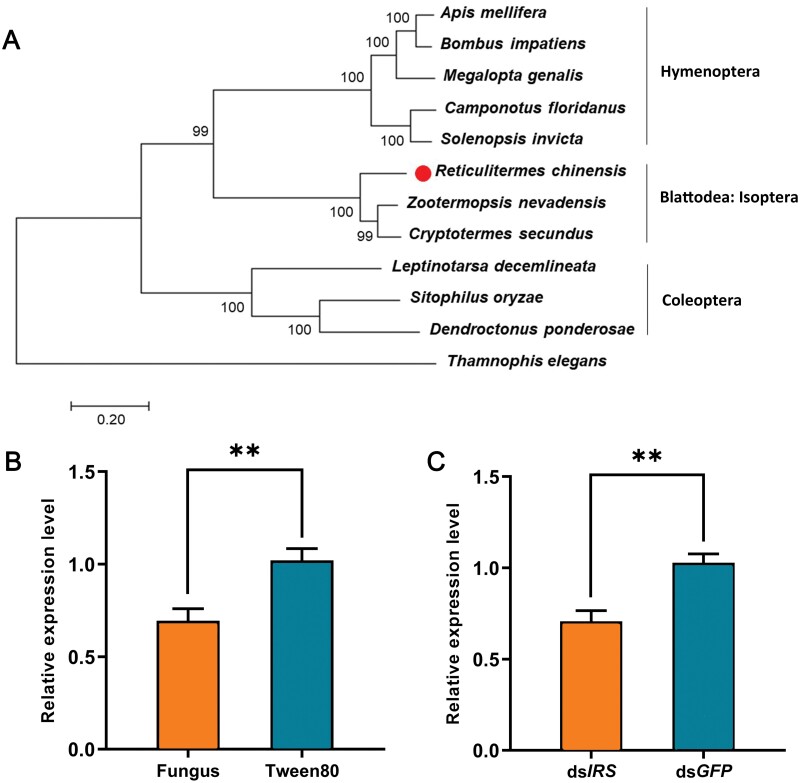
Cloning, expression, RNAi efficiency, and metabolic effect of IRS gene in *R. chinensis*. A) The phylogenetic tree of IRS from several insects. *R. chinensis* was marked with ‘●’. The scale bar indicates an average of 0.05 substitutions per site. B) IRS expression in the nestmates of the fungus-contaminated termites. C) IRS expression 3 days after ds*IRS* injection. The data are shown as mean + SEM (**, *P* < 0.01).

The phylogenetic trees showed that the amino acid sequences of *IRS* from different insect species are highly conserved (Fig. 1A). Moreover, the IRS proteins of *R. chinensis*, *Zootermopsis nevadensis*, and *Cryptotermes secundus* are in the same clade with *A. mellifera*, *Bombus impatiens*, *Megalopta genalis*, *Camponotus floridanus*, and *Solenopsis invicta*, which shows that they are more closely related. However, the IRS proteins of *Leptinotarsa decemlineata*, *Sitophilus oryzae*, and *Dendroctonus ponderosae* are not in the same branch with the 3 termite species, indicating that Coleoptera and termites are farther from each other.

One day after rearing together, the nestmates of the fungus-contaminated termites exhibited significantly lower *IRS* expression than the nestmates of the Tween 80-treated termites (Fig. 1B: *t* = 3.608, *df* = 16, *P* = 0.002), indicating that *IRS* may be involved in regulating active immunization against *M. anisopliae* in *R. chinensis*.

Three days after injections, *IRS* expression in the ds*IRS*-injected termites was significantly decreased compared to that in the control termites (Fig. 1C: *t* = 4.211, *df* = 16, *P* = 0.001).

### Effects of Suppressing *IRS* Gene on Metabolic Substances Levels of *R. chinensis*

**Fig. 2.**
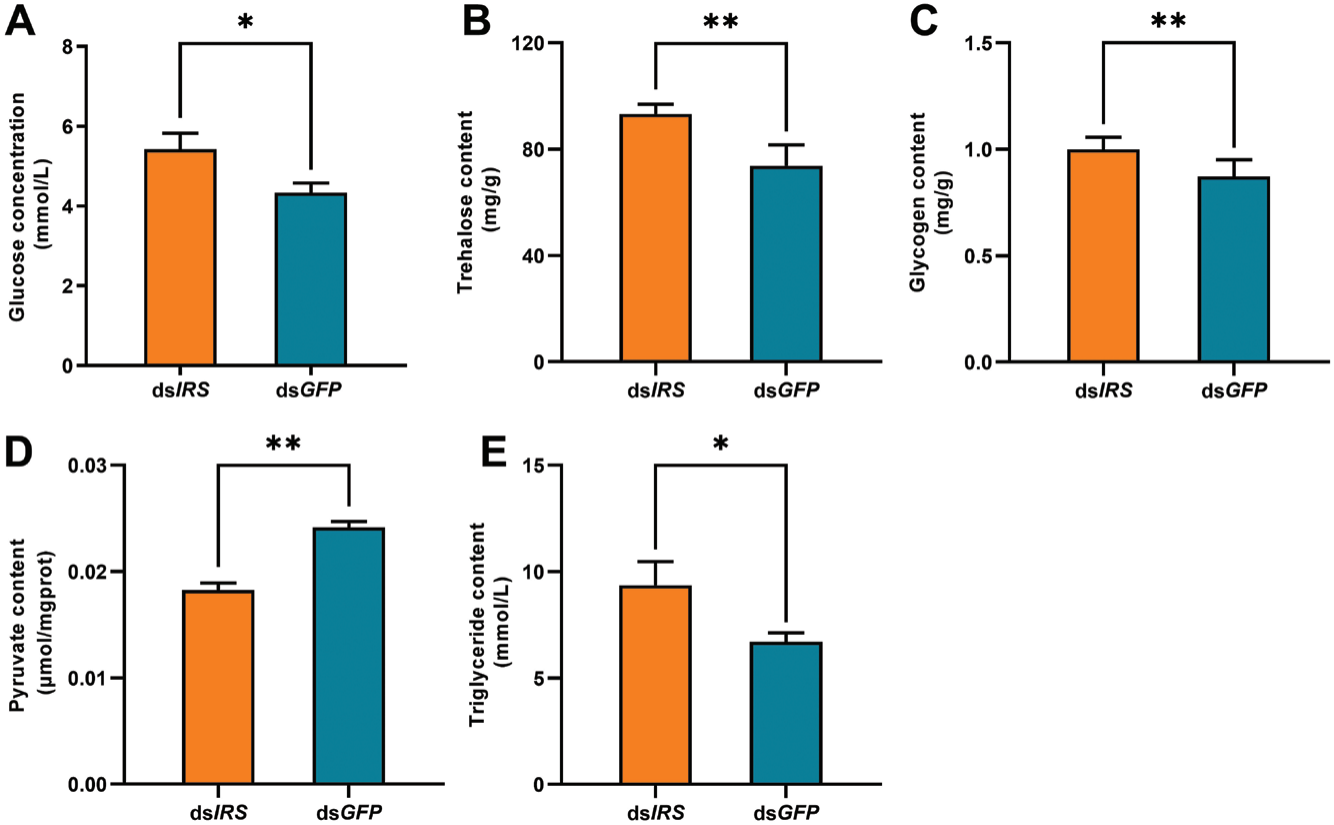
Effect of silencing IRS on the level of glucose, trehalose, and triglyceride in *R. chinensis*. A) Glucose level 3 days after ds*IRS* injection. B) Trehalose level 3 days after ds*IRS* injection. C) Glycogen level 3 days after ds*IRS* injection. D) Pyruvate level 3 days after ds*IRS* injection. E) Triglyceride level 3 days after ds*IRS* injection. The data are shown as mean + SEM (*, *P* < 0.01; **, *P* < 0.01).

After suppressing with *IRS* gene, glucose (Fig. 2A: *Z* = 2.252, *N* = 9, *P* = 0.024), trehalose (Fig. 2B: *Z* = 3.488, *N* = 9, *P* < 0.001), glycogen (Fig. 2C: *Z* = 2.608, *N* = 9, *P* = 0.009), and triglyceride (Fig. 2D: *Z* = 2.428, *N* = 9, *P* = 0.015) increased significantly in *R. chinensis*. In addition, suppressing *IRS* gene led to the decrease of pyruvate content in *R. chinensis* (Fig. 2E: *t* = 6.916, *df* = 16, *P* < 0.001).

### Effect of Suppressing *IRS* on the Grooming Behavior of *R. chinensis*

**Fig. 3.**
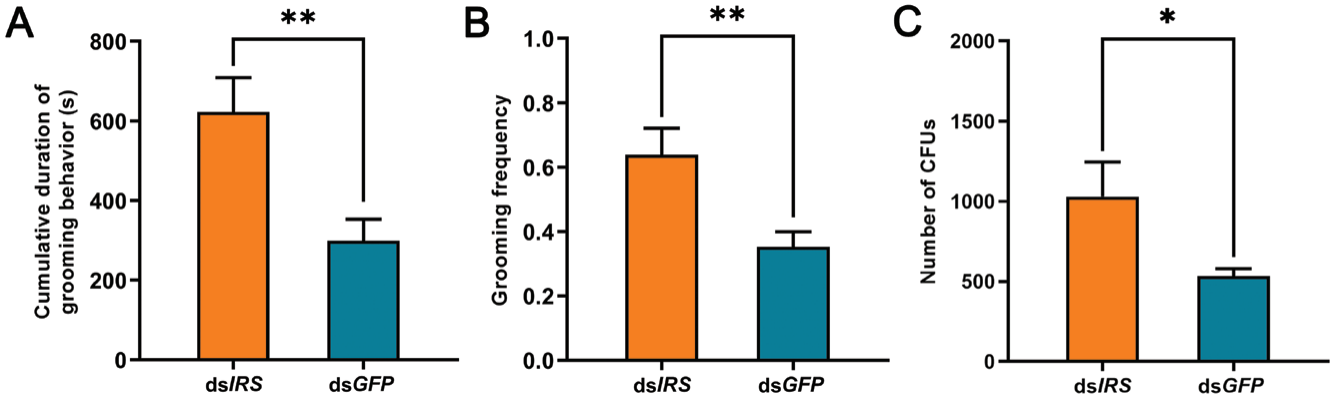
Effect of silencing IRS on grooming behavior of the nestmates of the fungus-contaminated termites. A) The cumulative duration of the grooming behavior. B) The grooming frequency. C) The number of colony-forming units of *M. anisopliae* in the gut. The data are shown as mean + SEM (*, *P* < 0.05; **, *P* < 0.01).

The cumulative duration of grooming behavior was significantly higher in ds*IRS*-injected nestmates of the fungus-contaminated termites than in the controls (Fig. 3A: *t* = 3.173, *df* = 16, *P* = 0.006). The grooming frequency was significantly higher in ds*IRS*-injected nestmates of the fungus-contaminated termites than in the controls (Fig. 3B: *t* = 3.033, *df* = 16, *P* = 0.008). The number of CFUs was significantly higher in ds*IRS*-injected nestmates of the fungus-contaminated termites than in the controls (Fig. 3C: *Z* = 1.984, *N* = 5, *P* = 0.047).

### Impact of *IRS* Knockdown on Antifungal Activity and Expressions of Apoptotic and Immune Genes

**Fig. 4.**
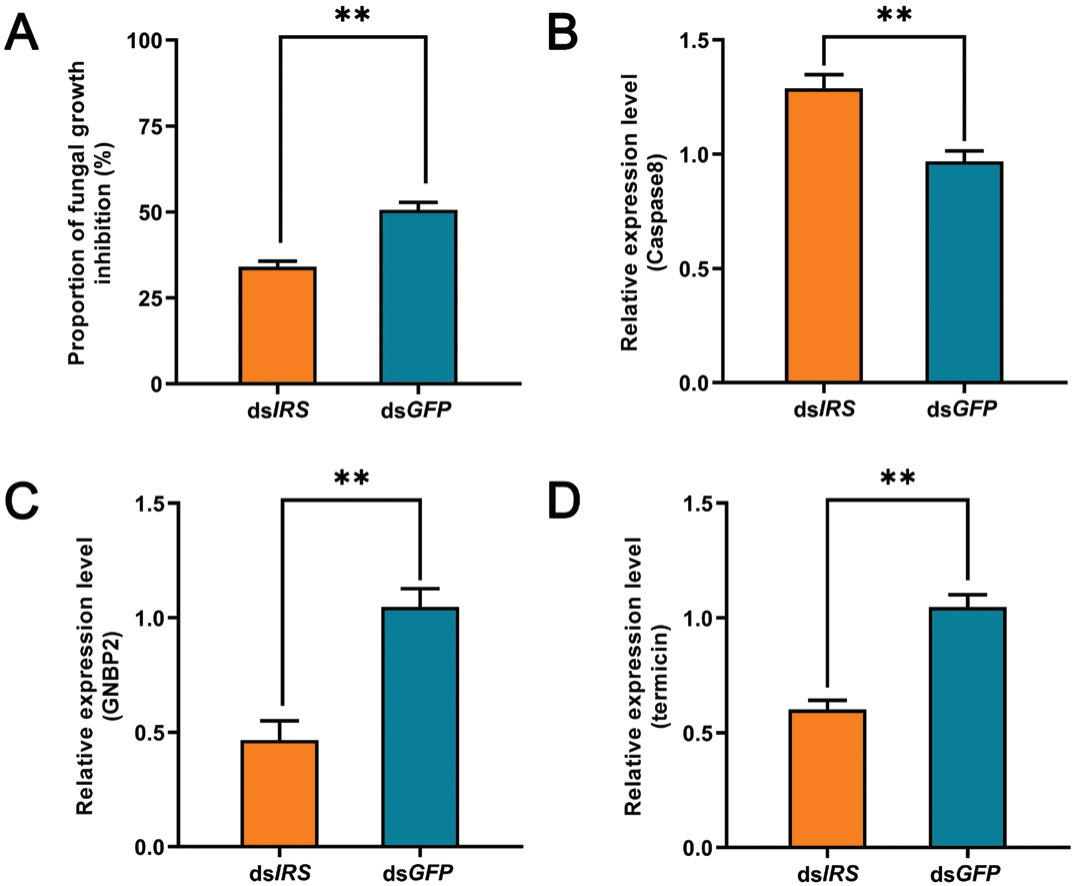
Impact of silencing IRS on antifungal activity and expressions of apoptotic and immune genes in the nestmates of the fungus-contaminated termites. A) The antifungal activity. B) The expression of the apoptotic gene *Caspase8*. C) The expression of the immune gene *GNBP2*. D) The expression of the immune gene *termicin*. The data are shown as mean + SEM (**, *P* < 0.01).

The antifungal activity of the ds*IRS*-injected nestmates of the fungus-contaminated termites was significantly lower than that of the control (Fig. 4A: *t* = 5.980, *df* = 16, *P* < 0.001). Additionally, *Caspase8* expression in the ds*IRS*-infected nestmates of the fungus-contaminated termites was significantly higher than that in the control (Fig. 4B: *t* = 4.175, *df* = 16, *P* = 0.001), but the expressions of *GNBP2* and *Termicin* in the ds*IRS*-infected nestmates of the fungus-contaminated termites was significantly lower than those in the controls (Fig. 4C: *t* = 5.024, *df* = 16, *P* < 0.001; Fig. 4D: *t* = 6.773, *df* = 16, *P* < 0.001, respectively).

**Fig. 5.**
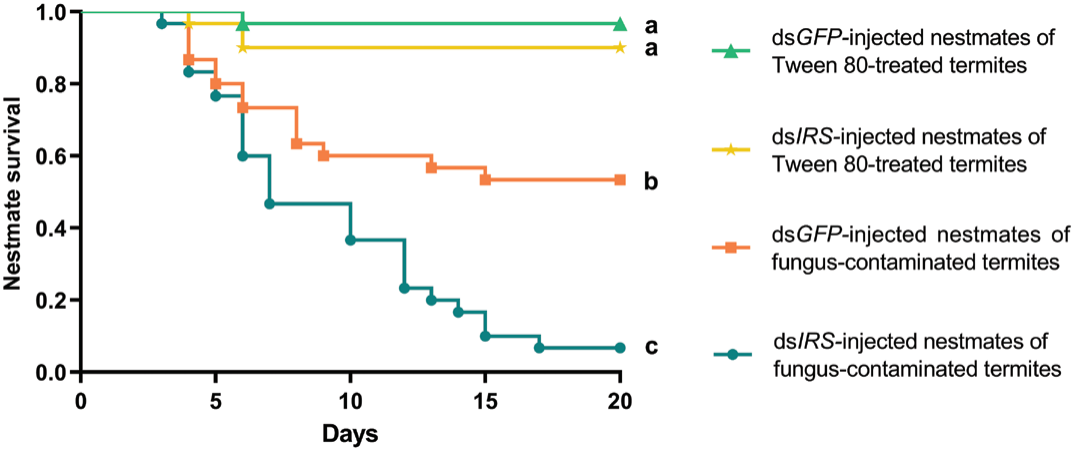
Influence of silencing IRS on the survival of the nestmates of the fungus-contaminated termites. Different letters indicate significant differences among the 4 groups (*P* < 0.01).

*Influence of IRS downregulation on the survival of termites*. Different treatments have a significant effect on nestmate survival (Wald test = 42.924, *df* = 1, *P* < 0.001), while different colonies have no significant effect on nestmate survival (Wald test = 3.450, *df* = 1, *P* = 0.063). The survival of the ds*IRS*-infected nestmates of the fungus-contaminated termites was significantly lower than that of the nestmates in the other groups (ds*IRS-*Fungus vs. ds*GFP-*Fungus: *χ*^2^ = 14.490, *P* < 0.001; ds*IRS-*Fungus vs. ds*IRS-*Tween 80: *χ*^2^ = 40.257, *P* < 0.001; ds*IRS-*Fungus vs. ds*GFP-*Tween 80: *χ*^2^ = 49.387, *P* < 0.001) (Fig. 5). The survival of the ds*GFP*-injected nestmates of the fungus-contaminated termites was significantly lower than that of the ds*GFP*-injected nestmates of the Tween 80-treated termites (ds*GFP-*Fungus vs. ds*GFP-*Tween 80: *χ*^2^ = 14.659, *P* < 0.001) (Fig. 5). The survival of the ds*GFP*-injected nestmates of the fungus-contaminated termites was significantly lower than that of the ds*IRS*-injected nestmates of the Tween 80-treated termites (ds*GFP-*Fungus vs. ds*IRS-*Tween 80: *χ*^2^ = 9.510, *P* = 0.002) (Fig. 5). There was no significant difference in survival between the ds*IRS-* and ds*GFP*-injected nestmates of the Tween 80-treated termites (ds*GFP-*Tween 80 vs. ds*IRS-*Tween 80: *χ*^2^ = 1.086, *P* = 0.297) (Fig. 5).

## Discussion

Insulin signaling plays an important role in maintaining metabolic homeostasis in organisms. Insect insulin regulates insects’ metabolism and immunity through signal transduction between cells (Xu et al. 2012, Spellberg and Marr 2015). The downregulated *IRS* gene increased the contents of glucose, trehalose, glycogen and triglyceride in termites, and decreased the content of pyruvate. The previous study showed that via ablation of the NSCs that produce insulin-like peptides, *Drosophila* had elevated levels of trehalose, glycogen, and lipids (Broughton et al. 2005). As another example, flies lacking the *IRS* Chico also exhibit elevated whole-body lipid levels (Böhni et al. 1999). Our results also prove this point, elevated sugars and lipids level in termites after suppressed *IRS* indicates that insulin signal is an important regulator of energy metabolism. Suppressing *IRS* may affect the homeostatic control of blood sugar in termites and causes termites to develop into a pathologically hyperglycemic and high triglyceride status. There is a tight coupling between immune function and metabolic function in the human (Konstantinos et al. 2015, Pearce 2021). The physiological and pathological processes of glucose and lipid metabolism disorders in humans involve the activation of the innate immune system, including the release of pro-inflammatory lipoproteins, disorders of antiinflammatory lipotroponin, insulin resistance and changes in monocyte chemokines (Reddy et al. 2019, Olona et al. 2021). The metabolic disorder of termites caused by injection of ds*IRS* may cause the imbalance of blood sugar of termites, then destroy the physiological immunity of termites, affect the immunity of termites to pathogenic fungi, and suppress with the active immunity.

Insulin signaling plays important role in driving behavioral immunity during active immunization in termite colonies. The interplay between immune response and insulin signaling has evolved as a means of the energy distribution during infection. The immune response reduces growth and nutrient storage in *Drosophila* by attenuating insulin signaling (DiAngelo et al. 2009). Inhibiting the insulin signaling pathway prolongs the survival of *Drosophila* against certain bacteria (McCormack et al. 2016). In our experiments, active immunization resulted in downregulation of the *IRS* gene (Fig. 1B), which shows that there is also an interaction between insulin signaling and active immunization in termites. Previous studies have found that insulin signaling can regulate caste-specific behaviors in social insects (Stern 2003, Erion and Sehgal 2013, Smykal and Raikhel 2015). In our study, we found that insulin signaling affected the hygiene behavior: The downregulated *IRS* enhanced grooming behavior of nestmates toward fungus-contaminated termites (Fig. 3A and B). When grooming, nestmates lick the pathogenic spores on the surface of infected termites (Liu et al. 2022). Enhanced grooming behaviors meant that more pathogenic spores were licked by the caregivers, and hence the downregulated *IRS* led to a larger number of CFUs in the gut of the nestmates of fungus-contaminated termite (Fig. 3C). These results indicate that *IRS* knockdown induces the nestmates of the fungus-contaminated termites to invest more in behavioral immunity during active immunization. However, once the conidial load exceeds the level that the caregivers can tolerate, they will face a great survival challenge.

*IRS* suppressing led to diminished physiological immunity and made nestmates more vulnerable to the social transferred fungal conidia. *Pseudomonas aeruginosa* can suppress host immunity by activating the DAF-2 insulin-like signaling pathway in *Caenorhabditis elegans* (Evans et al. 2008). *Chico* gene of *Drosophila* is a homologue of vertebrate *IRS1-4*, the functional loss of which reduced insulin signaling in *D. melanogaster* but increased the resistance to gram-positive and gram-negative bacterial infections (Libert et al. 2008). These suggest that insulin signaling and *IRS* play important roles in regulating immune responses. Normally fungus-exposed individuals led to low-level infections and enhanced the antifungal activity of nestmates due to social contact (grooming behavior) (Liu et al. 2015, Zhao et al. 2020). The previous studies showed that insulin signaling can modulate the innate immune response to bacterial pathogens, and also affect antimicrobial peptide activity (Spellberg and Marr 2015). In our study, suppressing *IRS* reduced the antifungal activity (Fig. 4A) and survival (Fig. 5) of nestmates grooming toward fungus-contaminated termites, indicating that their physiological immunity was weakened. Physiological immunity is an important component of active immunization, and weakening physiological immunity will eventually result in disruption of active immunization (Cremer 2019). Our suppressing *IRS* led to high-level of conidia load but diminished physiological immunity, which resulted in high risk of infection and mortality of the nestmates, and was more conducive to the epidemic outbreak in termite colonies.

*IRS* mediated active immunization by influencing the expression of immune and apoptotic genes in termites. *Termicin* is a defensin-like antimicrobial peptide from termites and shows strong antifungal activity. *GNBP2* can exhibit direct antifungal activity by breaking down β-1,3-glucan in the fungal cell wall, playing a direct role in termite antifungal defense (Kim et al. 2000, Lamberty et al. 2001, Xu et al. 2009, Hamilton and Bulmer 2012). Gene *GNBP2* and *termicin* are significantly upregulated in termites infected with pathogenic bacteria and fungi (Gao and Thompson 2015). The ds*IRS*-injected nestmates of the fungus-contaminated termite reduced their antifungal activity, possibly by inhibiting the upregulation of immune genes *GNBP2* and *termicin* (Fig. 4C and D). In addition, after suppressing *IRS*, the expression of apoptosis gene *caspase8* in the recipient termites was upregulated (Fig. 4B). The previous study showed that overexpressed *IRS* inhibited infection-induced apoptosis (Kaburagi et al. 2003, Shirakawa et al. 2013), while a small reduction in *IRS* also increased apoptosis (Lingohr et al. 2003, Ramocki et al. 2008). And hyperglycemia will induce apoptosis (Habib 2013), which is consistent with our previous results. We propose that suppressing IRS may lead to excessive apoptosis and synergistically disrupt active immunization in termites.

In summary, the knockdown of *IRS* gene resulted in more frequent and greater uptake of *M. anisopliae* spores by nestmate termites, which increased the chance of spore dispersal. In this process, suppressing *IRS* decreased the expressions of immune genes of the recipient termites, together with the upregulation of the cell apoptosis gene, weakened antifungal ability and therefore destroyed the physiological base of active immunization. Active immunity can reduce spore infection and protect termite colonies. However, the destruction of physiological immunity makes the high-frequency grooming behavior increase the infection amount, resulting in a burden on the nestmate termites. It was demonstrated that suppressing *IRS* gene impaired the active immunization of *R. chinensis* against *M. anisopliae*. Our study provides a novel technique to connect the insulin signaling pathway to immune response in termites. It suggests a potential strategy of using biological agents that combine EPF with ds*IRS* to efficiently manage termite pests in the field. Future research should focus on more efficient and precise techniques for delivering dsRNA into termite bodies to improve the effectiveness of *IRS* gene suppression. Exploring the potential synergies of combining *IRS* gene suppression with other biological or chemical control methods could lead to the development of integrated pest management strategies.

## Supplementary Material

ieae061_suppl_Supplementary_Tables_S1

ieae061_suppl_Supplementary_Tables_S2
