# Supplementary material for: Uncovering the function of insulin receptor substrate in termites’ immunity through active immunization
Source: J Insect Sci. 2024 Jul 3;24(4):1. doi: 10.1093/jisesa/ieae061 (PMC11221318; doi:10.1093/jisesa/ieae061)
Supplement: ieae061_suppl_Supplementary_Tables_S1 [file ieae061_suppl_supplementary_tables_s1.docx]

**Supplementary 1**

**Table S1 Primers used for cloning, qPCR and RNAi in this study**

| **Gene names** | **Primer sequences (5’→3’)** |
| --- | --- |
| Primers for cloning *IRS* gene | |
| *IRS*-F | GTCCCAATTCTCGTGGAAC |
| *IRS*-R | AGCATACGTGAAGGCCGATT |
| Primers for synthesizing dsRNA for RNAi^a^ | |
| ds*GFP*-F | GGATCCTAATACGACTCACTATAGGGCTTGAAGTTGACCTTGATGCC |
| ds*GFP*-R | GGATCCTAATACGACTCACTATAGGGTGGTCCCAATTCTCGTGGAAC |
| ds*IRS*-F | GGATCCTAATACGACTCACTATAGGG TGACTCCAGATCAGCCTGTG |
| ds*IRS*-R | GGATCCTAATACGACTCACTATAGGG TGAGCTCACCTTCTCCAGGT |
| Primers for quantitation of mRNA^b^ | |
| *HSP70*-F | GGAAACAGGACAACACCCAG |
| *HSP70*-R | GTGTCGTCAAATCTACGCCC |
| *β-actin*-F | AGCGGTCACTCATTCCCTTG |
| *β-actin*-R | ATTCCTGACGTACTGTCGCC |
| *IRS*-F | GAGGGAGCACAAGTCGAACA |
| *IRS*-R | ACGAGAAGAGTGCAGGTGTG |
| *Caspase8*-F | GGCCATGGGAGAGACAACTC |
| *Caspase8*-R | ATACTGGATGGCTCCGACCT |
| *GNBP2*-F | AAGCTGCACATCAAACCGTC |
| *GNBP2*-R | AGTTGATAACGCCGCTCGAT |
| *Termicin*-F | TCGTCTTTCTGGTCGTAGTG |
| *Termicin*-R | CAGTGGTGATAGAGATGATA |

^a^Nucleotide sequences corresponding to the T7 promoter region were underlined.

^b^The expression of *HSP70* and *β-actin* genes was used as the control.
