# Supplementary material for: Uncovering the function of insulin receptor substrate in termites’ immunity through active immunization
Source: J Insect Sci. 2024 Jul 3;24(4):1. doi: 10.1093/jisesa/ieae061 (PMC11221318; doi:10.1093/jisesa/ieae061)
Supplement: ieae061_suppl_Supplementary_Tables_S2 [file ieae061_suppl_supplementary_tables_s2.docx]

**Supplementary 2**

**Determination of metabolic substances**

**Table S2.1 Glucose content detection**

|  | **Blank Tube** | **Standard Tube** | **Sample Tube** |
| --- | --- | --- | --- |
| **Distilled Water (μL)** | 2.5 | / | / |
| **Standard Solution (μL)** | / | 2.5 | / |
| **Sample (μL)** | / | / | 2.5 |
| **Working Solution (μL)** | 250 | 250 | 250 |
| **Determination** | Gently shake the plate, incubate at 37℃ for 10 minutes, measure the absorbance (A) of each well at a wavelength of 505nm using the enzyme-linked immunosorbent assay(ELISA) reader. | | |
| **Calculation** | Glucose Content(mmol/L) = $\frac{\text{A}_{Sample}-\text{A}_{Blank}}{\text{A}_{Standard}-\text{A}_{Blank}}$×C_Standard_×N_Dilution_ | | |

^a^ C_Standard_ = 5.55mmol/L.

^b^ N_Dilution_: Dilution factor of sample.

**Table S2.2 Trehalose content detection**

|  | **Blank Tube** | **Standard Tube** | **Sample Tube** |
| --- | --- | --- | --- |
| **Reagent 1 (μL)** | 200 | / | / |
| **Standard Solution (μL)** | / | 200 | / |
| **Sample (μL)** | / | / | 200 |
| **Reagent 2 (μL)** | 800 | 800 | 800 |
| **Determination** | Mix well, cover tightly, then incubate in a 95℃ water for 10 minutes, followed by cooling under running water. After vortex mixing, transfer to a 1 cm path length cuvette and measure the absorbance values of each tube at 620nm using a spectrophotometer. | | |
| **Calculation** | Trehalose Content(mg/g) = $\frac{\text{A}_{Sample}-\text{A}_{Blank}}{\text{A}_{Standard}-\text{A}_{Blank}}$×C_Standard_×V_Extract_÷W_Sample_×N_Dilution_ | | |

^a^ C_Standard_ = 0.04mg/mL.

^b^ V_Extract_ = 1ml.

^c^ W_Sample_: Weight of sample.

^d^ N_Dilution_: Dilution factor of supernatant.

**Table S2.3 Glycogen content detection**

|  | **Blank Tube** | **Standard Tube** | **Sample Tube** |
| --- | --- | --- | --- |
| **Double-distilled Water (mL)** | 1.0 | / | 0.1 |
| **Standard Solution (mL)** | / | 1.0 | / |
| **Sample (mL)** | / | / | 0.9 |
| **Coloring Solution (mL)** | 2.0 | 2.0 | 2.0 |
| **Determination** | After thorough mixing, boil in boiling water for 5 minutes, then remove and cool. Zero the blank, measure the OD values of each tube at a wavelength of 620nm with a 1cm path length cuvette. | | |
| **Calculation** | Glycogen Content(mg/g) = $\frac{\text{A}_{Sample}}{\text{A}_{Standard}}$×M_Standard_×N_Dilution_×10÷1.11 | | |

^a^ M_Standard_ = 0.01mg/mL, Sugar content in standard tube.

^b^ N_Dilution_: Dilution factor of supernatant.

**Table S2.4 Pyruvate content detection**

|  | **Blank Tube** | **Standard Tube** | **Sample Tube** |
| --- | --- | --- | --- |
| **Double-distilled Water (mL)** | 0.1 | / | / |
| **Standard Solution (mL)** | / | 0.1 | / |
| **Sample (mL)** | / | / | 0.1 |
| **Reagent 1 (mL)** | 0.1 | 0.1 | 0.1 |
| **Reagent 2 (mL)** | 0.5 | 0.5 | 0.5 |
| **Determination** | After mixing well, incubate at in the 37℃ water for 10 minutes. | | |
| **Reagent 3 (mL)** | 2.5 | 2.5 | 2.5 |
| **Determination** | Allow to stand at room temperature for 5 minutes. Zero with double-distilled water, then measure the absorbance values of each tube at 505nm with a 1cm path length cuvette. | | |
| **Calculation** | Pyruvate Content(μmol/mgprot) = $\frac{\text{A}_{Sample}-\text{A}_{Blank}}{\text{A}_{Standard}-\text{A}_{Blank}}$×C_Standard_÷Cpr | | |

^a^ C_Standard_ = 0.2μmol/mL.

^b^ Cpr: Protein concentration of the homogenized tissue sample to be tested.

**Table S2.5 Triglyceride content detection**

|  | **Blank Tube** | **Standard Tube** | **Sample Tube** |
| --- | --- | --- | --- |
| **Distilled Water (μL)** | 2.5 | / | / |
| **Standard Solution (μL)** | / | 2.5 | / |
| **Sample (μL)** | / | / | 2.5 |
| **Working Solution (μL)** | 250 | 250 | 250 |
| **Determination** | Shake the plate well, incubate at 37℃ for 10 minutes, then measure the absorbance values of each well at a wavelength of 510nm using the ELISA reader. | | |
| **Calculation** | Triglyceride Content(mmol/L) = $\frac{\text{A}_{Sample}-\text{A}_{Blank}}{\text{A}_{Standard}-\text{A}_{Blank}}$×C_Standard_×N_Dilution_ | | |

^a^ C_Standard_ = 2.26mmol/L.

^b^ N_Dilution_: Dilution factor of sample.

**Table S2.6 Protein quantification detection**

|  | **Blank Tube** | **Standard Tube** | **Sample Tube** |
| --- | --- | --- | --- |
| **Distilled Water (μL)** | 10 | / | / |
| **Standard Solution (μL)** | / | 10 | / |
| **Sample (μL)** | / | / | 10 |
| **Working Solution (μL)** | 250 | 250 | 250 |
| **Determination** | Shake the plate well, incubate at 37℃ for 30 minutes, then measure the absorbance values of each well at a wavelength of 562nm using the ELISA reader. | | |
| **Calculation** | Triglyceride Content(μg/mL) = $\frac{\text{A}_{Sample}-\text{A}_{Blank}}{\text{A}_{Standard}-\text{A}_{Blank}}$× C_Standard_ ×N_Dilution_ | | |

^a^ C_Standard_ = 524μg/mL.

^b^ N_Dilution_: Dilution factor of sample.
